# The Association between *CDKAL1* Gene rs10946398 Polymorphism and Post-Transplant Diabetes in Kidney Allograft Recipients Treated with Tacrolimus

**DOI:** 10.3390/genes14081595

**Published:** 2023-08-07

**Authors:** Violetta Dziedziejko, Krzysztof Safranow, Mirosława Kijko-Nowak, Jerzy Sieńko, Damian Malinowski, Kamila Szumilas, Andrzej Pawlik

**Affiliations:** 1 Department of Biochemistry and Medical Chemistry, Pomeranian Medical University, 70-204 Szczecin, Poland; chrissaf@mp.pl; 2 Department of Nephrology, Transplantology and Internal Medicine, Pomeranian Medical University, 70-204 Szczecin, Poland; mirakijko@wp.pl; 3 Institute of Physical Culture Sciences, University of Szczecin, 70-453 Szczecin, Poland; jsien@poczta.onet.pl; 4 Department of Experimental and Clinical Pharmacology, Pomeranian Medical University, 70-204 Szczecin, Poland; damian.malinowski@pum.edu.pl; 5 Department of Physiology, Pomeranian Medical University, 70-204 Szczecin, Poland; kamila.szumilas@pum.edu.pl

**Keywords:** polymorphisms, post-transplant diabetes mellitus, tacrolimus, kidney transplantation

## Abstract

Post-transplant diabetes mellitus (PTDM) is a common complication that occurs in kidney transplant patients, increasing the risk of infection, cardiovascular disease and loss of graft function. Currently, factors that increase the risk of this complication are being sought, among them polymorphisms in genes that regulate carbohydrate metabolism and influence pancreatic β-cell function. The aim of this study was to evaluate the association of selected polymorphisms of genes affecting carbohydrate metabolism, such as *CDKAL1* rs10946398, *GCK* rs1799884, *GCKR* rs780094 and *DGKB/TMEM195* rs2191349, with the development of post-transplant diabetes in kidney transplant patients. This study included 201 Caucasian patients after kidney transplantation treated with tacrolimus. An association was observed between the *CDKAL1* rs10946398 gene polymorphism and PTDM. Among patients with PTDM, there was an increased prevalence of the CC genotype in the PTDM group compared to the group without PTDM. The chance of PTDM in those with the CC genotype was 2.60 times higher compared to those with the AC + AA genotypes (CC vs. AC + AA OR (95% CI): 2.60 (1.02–6.61), *p* = 0.040). Multivariate logistic regression analysis showed that advanced age and the CC genotype (rare homozygote) of *CDKAL1* rs10946398 were risk factors for the development of PTDM at 1 year after transplantation. There was no statistically significant association between *GCK* rs1799884, *GCKR* rs780094 or *DGKB/TMEM195* rs2191349 polymorphisms and the development of post-transplant diabetes mellitus in kidney transplant patients. The results of this study suggest that the *CDKAL1* rs10946398 CC genotype is associated with the increased risk of PTDM development in patients after kidney graft transplantation treated with tacrolimus.

## 1. Introduction

Kidney transplantation is the most effective treatment for kidney failure because it restores normal kidney function, resulting in a better quality of life and longer patient survival. Post-transplant diabetes mellitus (PTDM) is one of the most common complications of renal transplantation, worsening the prognosis of recipient survival and function of the transplanted organ. Patients with PTDM, compared to patients without PTDM, have a higher incidence of infection, episodes of acute rejection, a deterioration of graft function and faster graft loss, cardiovascular incidents (such as stroke and myocardial infarction), ischemic heart disease and death [1].

PTDM is most often found in the first year, especially in the first three months, after kidney transplantation. Its pathomechanism is similar to that of type 2 diabetes mellitus, but the exact pathogenesis remains unknown. In PTDM, both decreased insulin secretion by pancreatic β-cells and tissue insulin resistance were observed [2]. As shown, a number of factors lead to damage to pancreatic β-cells, resulting in reduced insulin secretion. There is also dysfunction of tissue glucose transporters, which is the cause of tissue insulin resistance and impaired glucose transport to tissues [3]. All of these mechanisms lead to elevated serum glucose levels. The development of PTDM is influenced by risk factors present before kidney transplantation, such as age of patient, metabolic syndrome, impaired glucose tolerance, obesity and a family history of type 2 diabetes, as well as the immunosuppressive treatment used [1,3,4]. The most potent diabetogenic drugs are calcineurin inhibitors, including tacrolimus. Tacrolimus has been shown to induce damage to pancreatic β-cells by enhancing their apoptosis, thereby reducing insulin secretion [5]. This drug also inhibits the transcription of the insulin gene and decreases the expression of glucose transporter GLUT4, resulting in increased insulin resistance [6]. Currently, we do not have biochemical indicators to effectively assess and predict the occurrence of post-transplant diabetes, so new biomarkers are being sought that will allow not only the earliest possible diagnosis and prediction of post-transplant diabetes but also the implementation of measures to prevent the development of the disease and apply timely treatment in the event of disorders.

Due to the similar pathogenesis of PTDM and type 2 diabetes, risk factors are currently being sought among the genetic polymorphisms associated with the development of type 2 diabetes [7]. Among them are polymorphisms in genes encoding enzymes involved in carbohydrate metabolism. To date, many of the genes responsible for the development of type 2 diabetes have also been shown to be associated with an increased risk of post-transplant diabetes [8].

Cyclin-dependent regulatory kinase subunit 5 (CDK5) regulatory subunit associated protein 1 like 1 (CDKAL1) is a protein encoded by the *CDKAL1* gene, located on the short arm of chromosome 6, consisting of 65 amino acids. CDKAL1 is involved in the pathogenesis of diabetes type 2 through impaired β-cell function [9].

Certain polymorphisms in the *CDKAL1* gene have been shown to be associated with a decrease in insulin sensitivity of the body’s tissues and an increased risk of type 2 diabetes [10]. The *CDKAL1* gene polymorphism rs10946398 was found to be associated with decreased insulin secretion by pancreatic β-cells [11].

Glucokinase (GCK) is an essential enzyme involved in glucose metabolism that belongs to the hexokinase family of isoenzymes. It is found in cells of the pancreas, liver, brain and gastrointestinal tract. It is encoded by the gene for glucokinase, which is located on chromosome 7. Glucokinase catalyses the phosphorylation reaction of glucose to glucose-6-phosphate in the cytoplasm. It is the first step in glucose metabolism [12]. The glucokinase gene rs1799884 polymorphism is associated with an increased risk of type 2 diabetes, abnormal fasting blood glucose, gestational diabetes and dyslipidaemia [13,14,15].

The glucokinase regulator (GCKR), otherwise known as glucokinase regulatory protein, (GKRP), is a 68 kDa protein containing 626 amino acids, which is formed in hepatocytes and is responsible for the binding and translocation of glucokinase, thus controlling both its activity and intracellular localisation. The gene encoding *GCKR* consists of 19 exons and is located on the short arm of chromosome 2 (2p23) [14]. The rs780094 polymorphism of the glucokinase regulator gene is associated with an increased risk of developing type 2 diabetes, gestational diabetes, abnormal fasting plasma glucose levels, dyslipidaemia and metabolic syndrome [15,16,17].

Diacylglycerol kinase β (DGKB or DAGK2) belongs to a family of enzymes that catalyses the reaction to convert diacylglycerol (DAG) to phosphatidic acid (PA) by using ATP as a phosphate source. It is a very small 13–15 kDa integral membrane protein encoded by the *DGKB* gene, located like the transmembrane protein 195 (*TMEM195)* gene on chromosome 7 (p21.2) [18]. Polymorphisms (for example rs2191349) in the *DGKB* and *TMEM195* intergenic region have been shown to influence the development of type 2 diabetes and obesity [19,20,21].

Due to the possible influence of genes that affect carbohydrate metabolism and are associated with an increased risk of type 2 diabetes as well as the diabetogenic effects of tacrolimus, the purpose of this study was to assess whether selected polymorphisms of these genes could affect the risk of PTDM in kidney transplant patients treated with tacrolimus [9,10,22,23]. We examined the association between *CDKAL1* rs10946398, *GCK* rs1799884, *GCKR* rs780094 and *DGKB/TMEM195* rs2191349 gene polymorphisms and the development of post-transplant diabetes in kidney transplant patients treated with tacrolimus.

## 2. Materials and Methods

### 2.1. Patients

The study group consisted of 201 Caucasian patients (108 male, 93 female) after kidney transplantation from a deceased donor. Patients in the whole group were treated with tacrolimus. Patients who had a diagnosis of diabetes mellitus (type 1 or type 2) before kidney transplantation (either as a cause of kidney disease or as a comorbidity) and patients with graft failure or death within the first month after transplantation were excluded from the study, and patients observed for less than 12 months were also excluded. Kidney transplants were performed at the Department of General and Transplant Surgery of Pomeranian Medical University. Immunosuppressive treatment of transplant patients included a standard three-drug therapeutic regimen with calcineurin inhibitors (tacrolimus (TAC), mycophenolate mofetil (MMF) and glucocorticosteroid (GCS). Tacrolimus dosing was started at 0.1 mg/kg body mass, with doses modified to maintain plasma concentrations between 10 and 12 ng/mL in the first month after transplantation and then between 8 and 10 ng/mL in subsequent months. Mycofenolate mofetil was administered at a dose of 2 g per day and prednisolone at a dose of 10–20 mg per day. Post-transplant diabetes was diagnosed in patients with glycated haemoglobin A1c levels above 6.5% and fasting glucose levels above 7.0 mmol/L, sustained for more than 3 months. The study was approved by the local ethics committee (BN-001/59/04), and written informed consent was obtained from all participants.

### 2.2. Genotyping

Genomic DNA was extracted from 1 mL of peripheral blood samples using a Genomic Mini AX Blood 1000 Spin kit (A&A Biotechnology, Gdynia, Poland) following the manufacturer’s protocol. Prior to isolation, blood samples were stored at −80 °C. DNA was subsequently standardised to equal concentrations of 20 ng/µL, based on spectrophotometric absorbance measurements (260/280 nm) (DeNovix DS-11 FX+ Spectrophotometer/Fluorometer, Wilmington, DE, USA) [24]. Testing for single-nucleotide polymorphisms was performed using TaqMan-type fluorescent hydrolysing probes with a real-time PCR instrument equipped with a 0.1 mL 96-well block (7500 Fast Real-Time PCR System instrument, Applied Biosystems, Wilmington, DE, USA). TaqMan^®^ Pre-Designed SNP Genotyping Assays were used (C__31635230_10, C___8304645_10, C___2862873_10, C__16153073_20; Applied Biosystems, Carlsbad, CA, USA).

### 2.3. Statistical Analysis

Statistical analysis of the obtained results was carried out using the statistical program STATISTICA 13. Compliance of the distributions of genotypes with the Hardy–Weinberg law (HWE) was evaluated using Fisher’s exact test. The chi-square test (χ2) was used to compare the distributions of genotypes and alleles between groups. Multivariate logistic regression analysis was used to evaluate independent factors associated with the risk of developing post-transplant diabetes during the year after kidney transplantation. *p* < 0.05 was used as the threshold for statistical significance.

## 3. Results

In this study, we examined patients after kidney allograft transplantation (108 male, 93 female). The mean age of the patients at the time of transplantation was 44.6 years (±13.2), and the mean body mass and BMI of the patients at the time of transplantation were 72.4 kg (±11.3) and 24.6 kg/m^2^ (±2.9), respectively. The follow-up period was 12 months after transplantation.

An association was observed between the *CDKAL1* rs10946398 gene polymorphism and PTDM. Among patients with PTDM, there was an increased prevalence of the CC genotype in the PTDM group compared to the group without PTDM. The risk of PTDM in individuals with the CC genotype was 2.60 times higher compared to those with the AC + AA genotypes (CC vs. AC + AA OR (95% CI): 2.60 (1.02–6.61), *p* = 0.040) (Table 1). PTDM was diagnosed in 15.1% of patients with AA genotype, in 15.7% of patients with AC genotype and in 32% of patients with CC genotype.

Next, we used multivariate logistic regression analysis to assess whether the CDKAL1 rs10946398 polymorphism is an independent risk factor for the occurrence of PTDM. Multivariate logistic regression analysis including sex, age, recipient BMI and *CDKAL1* rs10946398 polymorphism as independent variables in a recessive model showed that advanced age and the CC genotype (rare homozygote) of *CDKAL1* rs10946398 were risk factors for the development of PTDM at 1 year after transplantation in patients treated with tacrolimus (Table 2).

There was no statistically significant association between *GCK* rs1799884, *GCKR* rs780094 and *DGKB/TMEM195* rs2191349 polymorphisms and the development of post-transplant diabetes mellitus in kidney transplant patients treated with tacrolimus (Table 3 and Table 4). There were no statistically significant differences in the incidence of PTDM between patients with particular GCK rs1799884, GCKR rs780094 or DGKB/TMEM195 rs2191349 genotypes.

## 4. Discussion

The aim of this study was to evaluate the association between selected gene polymorphisms affecting carbohydrate metabolism and pancreatic β-cell function and the occurrence of post-transplant diabetes mellitus in patients treated with tacrolimus. PTDM occurs frequently after kidney transplantation and may be associated with graft loss, so factors that may increase the risk of this complication are being sought. Previous studies have shown that the pathophysiological changes leading to the development of PTDM are similar to those in type 2 diabetes mellitus [7,8,22,23]. It is believed that the risk factors leading to the occurrence of PTDM include factors associated with the development of type 2 diabetes mellitus, such as obesity, metabolic syndrome and a family history of type 2 diabetes mellitus. However, a very important influence on the occurrence of PTDM is the immunosuppressive therapy used, especially the use of the calcineurin inhibitor tacrolimus. Studies have shown that this drug exhibits strong diabetogenic effects due to its toxic effects against pancreatic β-cells, leading to reduced insulin production [5]. In animal models, tacrolimus has been shown to cause swelling of the cytoplasm of pancreatic β-cells and enhance their apoptosis [6]. It also reduces intracellular glucose transport by blocking its cellular transporters, resulting in insulin resistance [25].

Previous studies suggest that a number of factors influence the onset of PTDM, a key one being the diabetogenic effects of calcineurin inhibitors, especially tacrolimus [26]. The combination of the diabetogenic action of these drugs with other risk factors causes the development of PTDM. The goal of many current studies is to look for risk factors that may increase the risk of PTDM in patients treated with calcineurin inhibitors. Because of the similar pathophysiological background of PTDM and type 2 diabetes, the researchers focused on genetic polymorphisms that are associated with an increased risk of developing type 2 diabetes [27]. Among them are polymorphisms of genes affecting pancreatic β-cell function and carbohydrate metabolism. Therefore, in the current study we investigated the association between gene polymorphisms *CDKAL1* rs10946398, *GCK* rs1799884, *GCKR* rs780094 and *DGKB/TMEM195* rs2191349 and the development of PTDM. We demonstrated an association between *CDKAL1* rs10946398 gene polymorphism and PTDM. In contrast, we did not find an association between the other polymorphisms studied and the development of PTDM. The association of *CDKAL1* rs10946398 gene polymorphism and PTDM was confirmed by multivariate logistic regression analysis, where this polymorphism and recipient age were factors associated with the development of PTDM. To date, a number of genetic polymorphisms have been found to increase the risk of PTDM [27]. Previous studies indicated that the pathogenesis of PTDM is very complex and a number of factors influence the occurrence of this complication. These may include polymorphisms of genes that increase the risk of type 2 diabetes, affecting pancreatic β-cell function and carbohydrate metabolism. However, the main risk factor is the diabetogenic effect of immunosuppressive drugs from the calcineurin inhibitor group, which includes tacrolimus [26]. Probably there are many interactions between the action of tacrolimus and the metabolic pathways regulated by these genes. CDKAL1 plays a key role in post-translational modification and insulin secretion, as well as in the differentiation and development of pancreatic β-island cells. In mice lacking the *CDKAL1* gene, pancreatic β-cells produced reduced amounts of insulin after glucose stimulation [28]. CDKAL1 affects the proper differentiation and function of pancreatic β-cells and thus insulin secretion. CDKAL1 also regulates mitochondrial function and adipocyte differentiation [29]. It also participates in the development of inflammation in diabetes and affects the occurrence of diabetic complications [30]. Thus, it seems that there may be an interaction between the pathways modulated by CDKAL1 and the action of tacrolimus, which may lead to the occurrence of PTDM.

Many studies to date have confirmed the association between *CDKAL1* gene polymorphisms and the occurrence of type 2 diabetes and gestational diabetes [9,10,13]. The influence of *CDKAL1* gene polymorphisms on numerous parameters of carbohydrate and lipid metabolism, as well as on diabetes complications, has also been demonstrated. *CDKAL1* encodes a methyltransferase that regulates the CDK5 protein, which stimulates insulin production and other metabolic processes in pancreatic β-cells [31]. Previous studies have shown that the rs10946398 polymorphism of the CDKAL1 gene affects insulin secretion by pancreatic β-cells and is a risk factor for type 2 diabetes and gestational diabetes [9,10,13,14]. In addition, it has been shown that the rs10946398 polymorphism of the *CDKAL1* gene can also affect fasting glucose and insulin levels as well as levels of glycated haemoglobin HbA1c [11,32,33,34]. It may also have a role in diabetes management and the response to antidiabetic drugs [35].

The association between *CDKAL1* gene polymorphisms and PTDM has also been studied. Kang et al. found a statistically significant association between the rs10946398 polymorphism of the *CDKAL1* gene and PTDM in the Korean population [34]. In a meta-analysis by Benson et al. the C allele of the *CDKAL1* gene rs10946398 polymorphism was associated with an increased risk of diabetes after kidney transplantation (OR: 1.43, *p* = 0.006) [33]. Helvaci et al. found no statistically significant association between CDKAL1 gene rs7754840 polymorphism and new onset diabetes in kidney transplant recipients from the Turkish population [36].

We found no statistically significant association between the *GCK* rs1799884, *GCKR* rs780094 and *DGKB/TMEM195* rs2191349 polymorphisms and the risk of PTDM developing in patients treated with tacrolimus. These genes affect pancreatic β-cell function and insulin secretion, and their polymorphisms have been shown to be associated with the development of type 2 diabetes. Our results may suggest that there is no interaction between the pathways modulated by these genes and the effects of tacrolimus, which could explain the lack of association of the polymorphisms studied with PTDM. Since our work was limited by the number of patients, it is also possible that the effect of these genes on PTDM risk is so small that its detection would require studies on a much larger group of subjects.

PTDM is a metabolic complication that occurs in organ transplant patients, especially those treated with calcineurin inhibitors. The pathogenesis of PTDM is very complex and includes both impaired insulin secretion and tissue insulin resistance. Underlying this complication are factors affecting carbohydrate metabolism and pancreatic function, including genetic conditions. To date, many studies have considered the role of genetic polymorphisms as risk factors for the development of PTDM [8]. In particular, polymorphisms that have been confirmed to be associated with type 2 diabetes have been taken into account. It seems that the effect of a polymorphism of a particular gene on the development of PTDM may depend on the pathways that the gene regulates and whether these pathways are affected by the immunosuppressive drugs used, such as tacrolimus. It is likely that the effect of the drug used on certain metabolic pathways may lead to the development of PTDM. When assessing the risk of PTDM, it is important to consider a number of factors that may result in an increased probability of this complication. The results of the present study suggest that gene polymorphisms are one of the many factors that may affect the risk of developing PTDM and should be considered together with other known risk factors for the development of this complication. In particular, polymorphisms of genes involved in the regulation of glycaemia should be considered together with the diabetogenic effects of immunosuppressive pharmacotherapy and other risk factors for the development of PTDM. Due to the numerous complications of PTDM and its negative impact on the function of the transplanted kidney, there is a need to search for new markers predicting the risk of PTDM and to develop new immunosuppressive therapies with the least possible diabetogenic effect.

## 5. Conclusions

The results of this study suggest an association between *CDKAL1* gene rs10946398 polymorphism and post-transplant diabetes in kidney allograft recipients treated with tacrolimus. Patients with the *CDKAL1* rs10946398 CC genotype treated with tacrolimus had an increased risk of developing PTDM. The lack of evidence on the association of polymorphisms of other analysed genes involved in glycaemic regulation (*GCK*, *GCKR*, *DGKB/AGMO*) with the risk of post-transplant diabetes in kidney transplant recipients suggests that they do not play a key role in the development of this type of diabetes. Determining the exact role of genetic polymorphisms as risk factors for PTDM requires further multicentre studies.

## Figures and Tables

**Table 1:** Frequency distribution of genotypes and alleles of the rs10946398 polymorphism of the *CDKAL1* gene in the group of TAC-treated patients with post-transplant diabetes and the group without diabetes.

Genotype/Allele	No PTDM		PTDM		*p* ^^^		OR (95% CI)	*p* ^^^
	n	%	n	%				
*CDKAL1* rs10946398								
AA	90	54.22%	16	45.71%	0.12	CC + AC vs. AA	1.41 (0.68–2.92)	0.36
AC	59	35.54%	11	31.43%		CC vs. AC + AA	2.60 (1.02–6.61)	0.040
CC	17	10.24%	8	22.86%		CC vs. AA	2.65 (0.98–7.16)	0.049
						AC vs. AA	1.05 (0.46–2.42)	0.91
						CC vs. AC	2.52 (0.86–7.27)	0.081
A	239	71.99%	43	61.43%		C vs. A	1.61 (0.94–2.76)	0.079
C	93	28.01%	27	38.57%				

^ test χ^2^; *CDKAL1*—CDK5 regulatory subunit associated protein 1 like 1 gene; TAC—tacrolimus.

**Table 2:** Risk of developing PTDM within one year after transplantation in patients treated with tacrolimus (including *CDKAL1* rs10946398 in a recessive model).

Independent Variables	OR (95% CI)	*p*
Sex (male vs. female)	0.52 (0.23–1.17)	0.11
Recipient’s age (years)	1.04 (1.01–1.08)	0.019
Recipient’s BMI (kg/m^2^)	1.14 (0.99–1.30)	0.059
*CDKAL1* rs10946398 (CC vs. AC + AA) (recessive model)	3.01 (1.06–8.53)	0.037

*CDKAL1*—CDK5 regulatory subunit associated protein 1 like 1 gene, BMI—body mass index.

**Table 3:** Frequency distribution of genotypes and alleles of the rs1799884 polymorphism of the *GCK* gene and rs780094 of the *GCKR* gene in the group of TAC-treated patients with post-transplant diabetes and the group without diabetes.

Genotype/Allele	No PTDM		PTDM		*p* ^^^		OR (95% CI)	*p* ^^^
	n	%	n	%				
*GCK* rs1799884								
CC	125	75.30%	25	71.43%	0.50	TT + CT vs. CC	1.22 (0.54–2.75)	0.63
CT	37	22.29%	10	28.57%		TT vs. CT + CC	0.00 (-)	0.35
TT	4	2.41%	0	0.00%		TT vs. CC	0.00 (-)	0.37
						CT vs. CC	1.35 (0.60–3.07)	0.47
						TT vs. CT	0.00 (-)	0.30
C	287	86.45%	60	85.71%		T vs. C	1.06 (0.51–2.23)	0.87
T	45	13.55%	10	14.29%				
***GCKR* rs780094**								
CC	66	39.76%	9	25.71%	0.19	TT + CT vs. CC	1.91 (0.84–4.33)	0.12
CT	68	40.96%	20	57.14%		TT vs. CT + CC	0.87 (0.33–2.26)	0.77
TT	32	19.28%	6	17.14%		TT vs. CC	1.38 (0.45–4.20)	0.57
						CT vs. CC	2.16 (0.92–5.08)	0.074
						TT vs. CT	0.64 (0.23–1.74)	0.38
C	200	60.24%	38	54.29%		T vs. C	1.28 (0.76–2.14)	0.36
T	132	39.76%	32	45.71%				

^ test χ^2^; *GCK*—glucokinase gene; *GCKR*—glucokinase regulatory protein gene; TAC—tacrolimus.

**Table 4:** Frequency distribution of genotypes and alleles of the rs2191349 polymorphism of the *DGKB/TMEM195* gene in the group of TAC-treated patients with post-transplant diabetes and the group without diabetes.

Genotype/Allele	No PTDM		PTDM		*p* ^^^		OR (95% CI)	*p* ^^^
	n	%	n	%				
*DGKB/TMEM195* rs2191349								
TT	47	28.31%	13	37.14%	0.48	GG + GT vs. TT	0.67 (0.31–1.44)	0.30
GT	78	46.99%	16	45.71%		GG vs. GT + TT	0.63 (0.24–1.62)	0.34
GG	41	24.70%	6	17.14%		GG vs. TT	0.53 (0.18–1.52)	0.23
						GT vs. TT	0.74 (0.33–1.68)	0.47
						GG vs. GT	0.71 (0.26–1.96)	0.51
T	172	51.81%	42	60.00%		G vs. T	0.72 (0.42–1.21)	0.21
G	160	48.19%	28	40.00%				

^ test χ^2^; *DGKB/TMEM195*—diacylglycerol kinase β/transmembrane protein 195 gene; TAC—tacrolimus.

## Data Availability

The data that support the findings of this study are available upon reasonable request from the corresponding author.
